# Monitoring Distribution Dynamics of EV RNA Cargo Within Recipient Monocytes and Macrophages

**DOI:** 10.3389/fcimb.2021.739628

**Published:** 2022-01-26

**Authors:** Daniel Alfandari, Hila Ben Ami Pilo, Paula Abou Karam, Osnat Dagan, Carine Joubran, Ron Rotkopf, Neta Regev-Rudzki, Ziv Porat

**Affiliations:** ^1^ Department of Biomolecular Sciences, Faculty of Biochemistry, Weizmann Institute of Science, Rehovot, Israel; ^2^ Bioinformatics Unit, Life Sciences Core Facilities, Weizmann Institute of Science, Rehovot, Israel; ^3^ Flow Cytometry Unit, Life Sciences Core Facilities, Weizmann Institute of Science, Rehovot, Israel

**Keywords:** *Palsmodium falciparum*, extracellular vesicles (EVs), imaging flow cytometry (IFC), parasite, uptake

## Abstract

Extracellular vesicles (EVs) are produced by across almost all the living kingdoms and play a crucial role in cell-cell communication processes. EVs are especially important for pathogens, as *Plasmodium falciparum (Pf)* parasite, the leading causing species in human malaria. Malaria parasites are able to modulate the host immune response from a distance *via* delivering diverse cargo components inside the EVs, such as proteins and nucleic acids. We have previously shown that imaging flow cytometry (IFC) can be effectively used to monitor the uptake of different cargo components of malaria derived EVs by host human monocytes. Here, we take this approach one step further and demonstrate that we can directly investigate the dynamics of the cargo distribution pattern over time by monitoring its distribution within two different recipient cells of the immune system, monocytes vs macrophages. By staining the RNA cargo of the vesicles and monitor the signal we were able to evaluate the kinetics of its delivery and measure different parameters of the cargo’s distribution post internalization. Interestingly, we found that while the level of the EV uptake is similar, the pattern of the signal for RNA cargo distribution is significantly different between these two recipient immune cells. Our results demonstrate that this method can be applied to study the distribution dynamics of the vesicle cargo post uptake to different types of cells. This can benefit significantly to our understanding of the fate of cargo components post vesicle internalization in the complex interface between pathogen-derived vesicles and their host recipient cells.

## Introduction

Extracellular vesicles (EVs) are lipid bilayer nanoparticles that are secreted by cells to deliver cargo components into target cells (van der Pol et al., 2012, Mathieu et al., 2019). They are mainly classified into two main groups, according to their origin: microvesicles (200-1000 nm in diameter) are shed from the plasma membrane, while exosomes (30-200 nm in diameter) originate from invagination of multi-vesicular bodies (MVB), and are released to the extracellular space by fusion with the plasma membrane (Raposo and Stoorvogel, 2013; Montaner et al., 2014; van der Pol et al., 2014). EV secretion is an evolutionary conserved mechanism, used by many different organisms (Montaner et al., 2014; Schorey et al., 2015; Ofir-Birin et al., 2017; Mathieu et al., 2019), mediating intercellular communication by the delivery of a wide range of bioactive components, including proteins, lipids, glycans, DNA and RNA (Marcilla et al., 2014; Pelissier Vatter et al., 2021; Couch et al., 2021).

In particular, EVs play a crucial role in the complex life cycle of many pathogens, and mediate processes such as pathogen growth and development, virulence factor transfer and immune responses manipulation (Montaner et al., 2014; Schorey et al., 2015; Ofir-Birin and Regev-Rudzki, 2019). One such prominent example is the deadly malaria parasite in humans, *Plasmodium falciparum* (*Pf*). During their life-cycle, *Pf* parasites secrete EVs while growing inside human red blood cells (RBCs), which deliver multiple cargo components (Regev-Rudzki et al., 2013; Mantel et al., 2016; Sisquella et al., 2017; Ye et al., 2018; Avalos-Padilla et al., 2021; Dekel et al., 2021; Ofir-Birin et al., 2021). These EVs modulate different host cells by promoting parasitic invasion (Dekel et al., 2021), endothelial cell modulation (Mantel et al., 2016) and immune cell alteration (Mantel et al., 2013; Sisquella et al., 2017; Ye et al., 2018; Ofir-Birin et al., 2021). For instance, it was shown that *Pf*-derived EVs deliver parasitic genomic DNA (gDNA), recognized by the STING-dependent cytosolic DNA sensing pathway in recipient monocytes (Sisquella et al., 2017). By activating this pathway, the parasite is able to modulate host gene expression from a distance, and to induce transcription of Type-I IFN dependent genes (Sisquella et al., 2017). In addition, it was demonstrated that uptake of *Pf*-derived EVs into host primary Natural Killer (NK) cells serves as a delivery mode of parasitic RNA into the cytosol of these cells (Ye et al., 2018). *Pf*-derived EVs have also been demonstrated to be efficiently internalized by macrophages, inducing a strong inflammatory response, including activation of pro-inflammatory cytokines IL-6, IL-12 and IL-1β and the anti-inflammatory cytokine IL-10 in a dose-dependent manner (Mantel et al., 2013).

In order to study the mechanisms underlying EV uptake, several endocytic inhibitors have been used (Mulcahy et al., 2014; Anakor et al., 2021); still, the precise molecular mechanism that regulates EV uptake followed by the cargo distribution within target cell, is unknown (Mulcahy et al., 2014; Mathieu et al., 2019). Moreover, in some systems, as for *Pf*-derived EVs, one of the main challenges in studying EV uptake is the lack of commercially available antibodies specific for malaria-derived antigens (Dekel et al., 2020). In order to overcome this, we have previously developed a method that allows us to label EVs without the need for antibodies but by labeling other cellular components (Dekel et al., 2020). By doing so, we could monitor different EV populations according to their distinct cargo components, and the cargo distribution inside recipient cells post uptake (Dekel et al., 2020).

One large-scale imaging approach implemented impressively in the EV field is the use of imaging flow cytometry (IFC) (Fendl et al., 2016; Lannigan and Erdbruegger, 2017; Ofir-Birin et al., 2018). Combining the speed and high-throughput of conventional flow cytometry, with the information-rich imagery of microscopy, this technique allows a rapid acquire of high-quality multispectral images (Ortyn et al., 2006; Erdbrügger et al., 2014; Vorobjev and Barteneva, 2016). Using IFC enables the measurement of fluorescence levels, cellular localization and co-localization of different markers, and pixel distribution. Due to the nano-size of EVs, which falls within the range of electronic noise, a big advantage of IFC is its ability measure single pixel intensities and thus detect fluorescent particles that are smaller than the diffraction limit (Ortyn et al., 2006; Erdbrügger et al., 2014; Clark, 2015; Vorobjev and Barteneva, 2016). Indeed, it was previously shown that IFC can be used as an accurate large-scale method for tracking the dynamics of the uptake of individual types of cargo components (RNA, DNA, proteins, and lipids) (Sisquella et al., 2017; Ofir-Birin et al., 2018).

Here, we took this approach one step further, and monitored by live uptake assay the kinetics distribution of the signal of RNA cargo of *Pf*-derived EVs within two different recipient cells of the human host immune system, monocytes vs macrophages. By staining the RNA cargo of the vesicles, we were able to directly track the cargo’s internalization over time and measure the dynamics of the RNA distribution inside human monocytes and macrophages. We examined three different parameters post uptake of the vesicles to the recipient cells over the course of 1 hour. Surprisingly, we observed significant differences in the dynamics of the RNA cargo distribution within monocytes vs macrophages, which might suggest a distinct roles for the RNA cargo post uptake to the target cells. While in the monocytes the pattern of the cargo distribution is more diffusive and the max contour localizes towards the center of the cell, in the macrophages the pattern is more concentrated and localizing towards the cell perimeter, over the course of time. These data demonstrate that IFC can serve as a powerful approach to directly follow the distribution dynamics of EV cargo components post uptake into different target cells. This could pave the way to a not just better understanding of the EV’s mediated communication, but also may provide new insights on the functions of distinct cargo component.

## Materials And Methods

### Cell Culture

#### Human Malaria Parasites (*Plasmodium falciparum*)

NF54 *Pf* WT cells [generously provided by Malaria Research Reference Reagent Resource Center (MR4)] were grown in pooled donor RBCs provided by the Israeli blood bank (Magen David Adom blood donations in Israel) at 4% hematocrit and incubated at 37°C in gas mixture of 1% O_2_, 5% CO_2_ in N_2_. Parasites were maintained in complete RPMI medium pH 7.4, 25 mg/ml HEPES, 50 μg/ml hypoxanthine, 2 mg/ml sodium bicarbonate, 20 μg/ml gentamycin, and 0.5% AlbumaxII. *P. falciparum* cultures were tested for mycoplasma once a month using a commercial kit: MycoAlert Plus kit (Lonza). Growth was monitored using methanol fixed Giemsa-stained blood smears (Sisquella et al., 2017).

#### Human Monocytes (THP-1 Cells)

THP-1 cell line was cultured as previously described (Unterholzner et al., 2010). In brief, cells were grown in complete RPMI 1640+, L-glutamine, and 10% FBS, in a humidified incubator at 37°C, with 5% CO2. Cells were tested for mycoplasma once a month using commercial kit: MycoAlert Plus kit (Lonza).

#### Human Macrophages

4×10^6^ THP-1 cells were plated on a 10 ml plate and differentiated into macrophages by 72 hours incubation with 100nM phorbol 12-myristate 13-acetate (PMA, Sigma, P8139), followed by 48 hours incubation in RPMI medium. Cells were passaged to a new plate, using Trypsin EDTA solution C (03-054-1), in case of very high confluency prior to the experiment. In order to determine the percentages of monocyte’s differentiation to macrophages, untreated cells and cells treated with PMA as was previously shown (Baxter et al., 2020) were compared for different properties. Briefly, cells were incubated for 10 minutes on ice with human Fc block (BD Pharmingen) in order to block nonspecific sites. The cells were resuspended in FACS buffer, and immunofluorescence staining for CD14 was performed with mouse anti-human CD14 APC (1:100 dilution, Biogems) in the dark for 30 minutes on ice. Treated cells were analyzed by IFC. At least 5×10^4^ cells were collected from each sample and data were analyzed using the manufacturer’s image analysis software (IDEAS 6.3; Amnis Corp).

### EV Production

Media of high parasitemia (≥5%) *Pf-*trophozoite blood stage culture was collected. Prior to media collection, cultures were tightly synchronized by using 5% sorbitol, according to standard protocols (Sisquella et al., 2017). EVs purification was performed as previously described (Dekel et al., 2021). Briefly, media of *Pf-*infected RBC culture was spun down at 1,500 rpm for 5 minutes. The remaining cells were cleared by an additional centrifugation at 1,500 rpm for 5 minutes, followed by a centrifugation at 3,000 rpm for 10 minutes. To eliminate cell debris, the media was then centrifuged at 10,500 rpm for one hour at 4°C. The supernatant was filtered through a 450 nm pore filter and EVs were concentrated using a Vivacell^®^ 100 with a 100kDa cut-off, according to the manufacturer’s protocol. EVs were stained using Thiazole Orange (TO) for RNA staining at a dilution of 1:1,000 and left in 37°C for 30 minutes. Unstained EVs and untreated monocytes and macrophages were used as controls. An overnight ultracentrifugation step at 4°C and 37,000 rpm using a Beckman OPTIMA90X ultracentrifuge with a TI70 rotor, as previously described (Coleman et al., 2012), then followed, in order to pellet the exosomes. Finally, the pellet was carefully suspended in PBS (Calcium and Magnesium free) for further analysis.

### NTA Analysis for Vesicle Size and Concentration

Analysis was conducted using NanoSight NS300 nanoparticle tracking instrument (NTA) (Filipe et al., 2010). This device monitors the Brownian motion of nanoparticles of 10-1,000 nm size, using light scattering. The software then calculates the concentration and size distribution of the nanoparticles. During these measurements, each EV sample (in a 1:1,000 dilution) was measured five times for 60 seconds.

### Live Uptake Kinetic Assay

THP-1 cell (human monocytes) line and macrophages differentiated from THP-1 cells (Baxter et al., 2020) were cultured overnight in RPMI1640+ l-glutamine (Biological Industries Ltd., Beit Ha’Emek, Israel) and 10% FBS (Biological Industries Ltd., Beit Ha’Emek, Israel). For live uptake kinetic assay, 1-2×10^6^ monocytes or macrophages were stained with Hoechst (4 µM, Life Technologies) at a dilution of 1:8,000 and left in the incubator for 30 minutes, followed by 2 washes with PBS (Calcium and Magnesium free). Cells were kept on ice prior for incubation with TO stained EVs and run immediately by IFC for 1 hour.

### Multispectral IFC Analysis

Cells were imaged using a multispectral IFC (ImageStreamX mark II imaging flow-cytometer: Amnis Corp, Seattle, WA, Part of Luminex). *Pf*-derived EVs, labeled with TO were added to host cells. At least 5×10^4^ cells were collected from each sample and data were analyzed using the manufacturer’s image analysis software (IDEAS 6.3; Amnis Corp). Monocytes and macrophages were gated for single cells, using the area and aspect ratio features, and for focused cells, using the Gradient RMS feature, as previously described (Fendl et al., 2016). Cropped cells were further eliminated by plotting the cell area of the bright field image against the Centroid X feature (the number of pixels in the horizontal axis from the left corner of the image to the center of the cell mask). Vesicle internalization was evaluated using several features, including the intensity (the sum of the background-subtracted pixel values within the masked area of the image) and the max pixel (the largest value of the background-subtracted pixel). The area of highest intensity pixels was calculated by the Area feature (sum of pixels within the image mask) using the Threshold_60 mask (selects the 60% top intensity pixels) on the Thiazole Orange staining (Ch02). The localization of the peak intensity was quantified using the Max Contour Position feature (the location of the contour in the cell that has the highest intensity concentration mapped to a number between 0 and 1, with 0 being the object center and 1 being the object perimeter). It is invariant to object size and can accommodate localized intensity concentrations. For quantification of apoptotic cells, single, focused cells were plotted for the contrast of the bright field channel vs. the area of the 50% highest intensity pixels of the Hoechst staining (defined by the Threshold_50 mask). Cells with high contrast and low area (condensed) of the DNA staining were considered apoptotic (Wortzel et al., 2017).

### THP-1 Uptake and Organelle Fluorescence Staining

THP-1 cells were incubated with labeled EVs for 5 and 45 minutes and were fixated in 4% PFA2% sucrose for 10 minutes at room temperature and washed with PBS (Calcium and Magnesium free). Cells were incubated with 5% BSA in PBS for 30 minutes at room temperature to block nonspecific sites. The treated cells were resuspended in PBS, and immunofluorescence staining of the organelles was performed as follows. First, cells were incubated over night at 4°C with the following primary antibodies: mouse anti-NPM1 (1:500 dilution, Sigma-Aldrich) for the Nucleolus sub-cellular localization control, mouse anti-LAMP2 (1:100 dilution, DSHB) for the Lysosome sub-cellular localization, rabbit anti-Giantin (1:500 dilution, Abcam) for the Golgi sub-cellular localization. The samples were followed by incubation with Alexa Fluor 647-labeled donkey anti-rabbit IgG (H+L) (1:500 dilution, Life Technologies) and with Cy3 labeled donkey anti-mouse IgG (H+L) (1:500 dilution, Jackson) secondary antibodies in dark for 1 hour. Second, in addition to staining the nucleolus, THP-1 cells were stained with Hoechst. Lastly, cells were analyzed by IFC.

### Statistical Analysis

Cell types were compared with a linear mixed effects model, with cell type and time as fixed factors, and sample ID as a random factor. Statistics were done in R, v. 4.0.4, using the package ‘lmerTest’. For calculating changes relative to the starting point, a log2-fold change was calculated per each point, relative to its own sample’s average value of the first 1.5 minutes. Grey area around the lines represents 95% confidence intervals.

## Results

### Monitoring the Kinetic of *Pf*-Derived EV Uptake Into Different Subsets of Immune Cells; Macrophages and Monocytes

We directed efforts to establish a large-scale research tool to characterize the interface between pathogen-derived EVs and their host-recipient cells. We took an advantage of a live-uptake assay and antibody-free labeling previously established methods (Erdbrügger et al., 2014; Clark, 2015; Dekel et al., 2020), to monitor the vesicle cargo distribution in host immune recipient cells, by using IFC. Using this advanced method of measuring live uptake kinetic allowed us to follow visually the simultaneous delivery of different cargo components into recipient monocytes (Sisquella et al., 2017; Ofir-Birin et al., 2018). This led us to investigate the intriguing question-what is the fate of the EV’s cargo (e.g. RNA) dynamic distribution post internalization into different host target cells, specifically different subsets of immune cells, monocytes vs macrophages. In order to validate a significant differentiation of monocytes into macrophages, naïve THP-1 cells were treated with phorbol 12-myristate 13-acetate (PMA) as previously described (Baxter et al., 2020). Untreated monocytes and monocyte-derived macrophages were then stained using anti-CD14 cell surface marker antibody (Daigneault et al., 2010; Aldo et al., 2012; Starr et al., 2018) and were analyzed using IFC (**Supplementary Figure 1**). As demonstrated, following PMA treatment, macrophages were significantly bigger and showed an increased CD14 expression on their surface, thus demonstrating a successful differentiation of monocytes into macrophages (**Supplementary Figure 1**).

**Figure 1:**
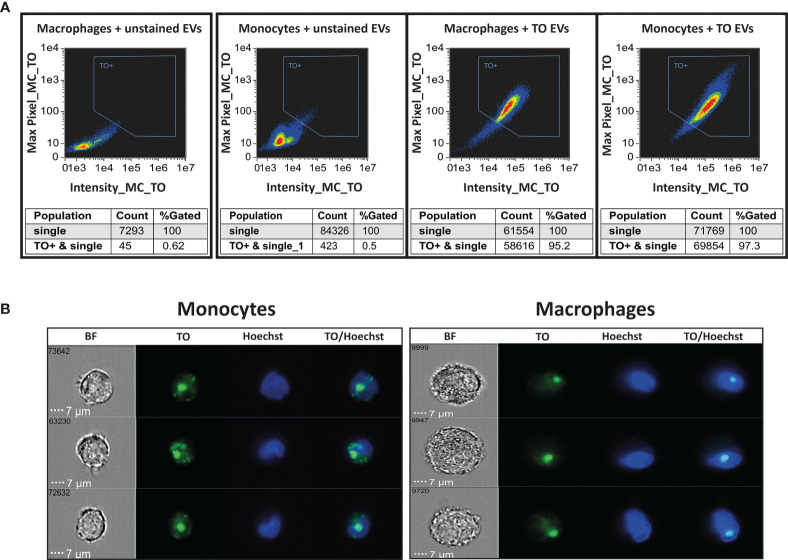
Monitoring uptake of *Pf*-derived EVs into monocytes and macrophages. *Pf*-derived EVs were labeled by Thiazole orange (TO) and their uptake into monocytes (THP-1 cells) or macrophages (differentiated from THP-1 cells) was measured for 1 hour. **(A)** Graphs show the percentage of TO-labeled EV-positive cells after 1 hour, gated according to unlabeled EVs. Tables below the pictures show the percentages of TO positive cells. Representative results from at least three independent experiments are shown. Host cells incubated with unstained EVs were used as controls (left panels). **(B)** Signal detected by IFC from three individual representative recipient cells of monocytes and macrophages, 1 hour post uptake. BF-bright field, TO-thiazole orange, Hoechst-nuclear dye.

By staining the RNA cargo of the vesicles, we were able to directly track the RNA cargo’s internalization over time and measure the dynamics of its distribution inside monocytes and macrophages. EVs were purified from *Pf*-iRBCs culture (≥5% parasitemia) and stained using Thiazole Orange (TO) RNA dye (Sisquella et al., 2017). EV levels were counted by NTA measurement, **Supplementary Figure 2**. THP-1 cells or THP-1 cells which were differentiated into macrophages by treatment with PMA, were introduced to the RNA-labeled EVs. As controls, THP-1 cells and differentiated macrophages were incubated with unlabeled *Pf*-derived EVs. The derived fluorescent signal was read continuously by IFC for 1 hour of uptake. A trend line was calculated by the statistical software R, using the “ggplot2” package (Wickham, 2008). The signals were compared with the acquisition of unlabeled EVs (**Figure 1A**). The EV uptake into monocytes and macrophages occurs rapidly; 10 minutes post co-incubation most of the monocytes (>90%) stained positive for the RNA-cargo (TO dye). Representative cell images from three recipient monocytes or macrophages after 1 hour post uptake are shown in **Figure 1B**.

**Figure 2:**
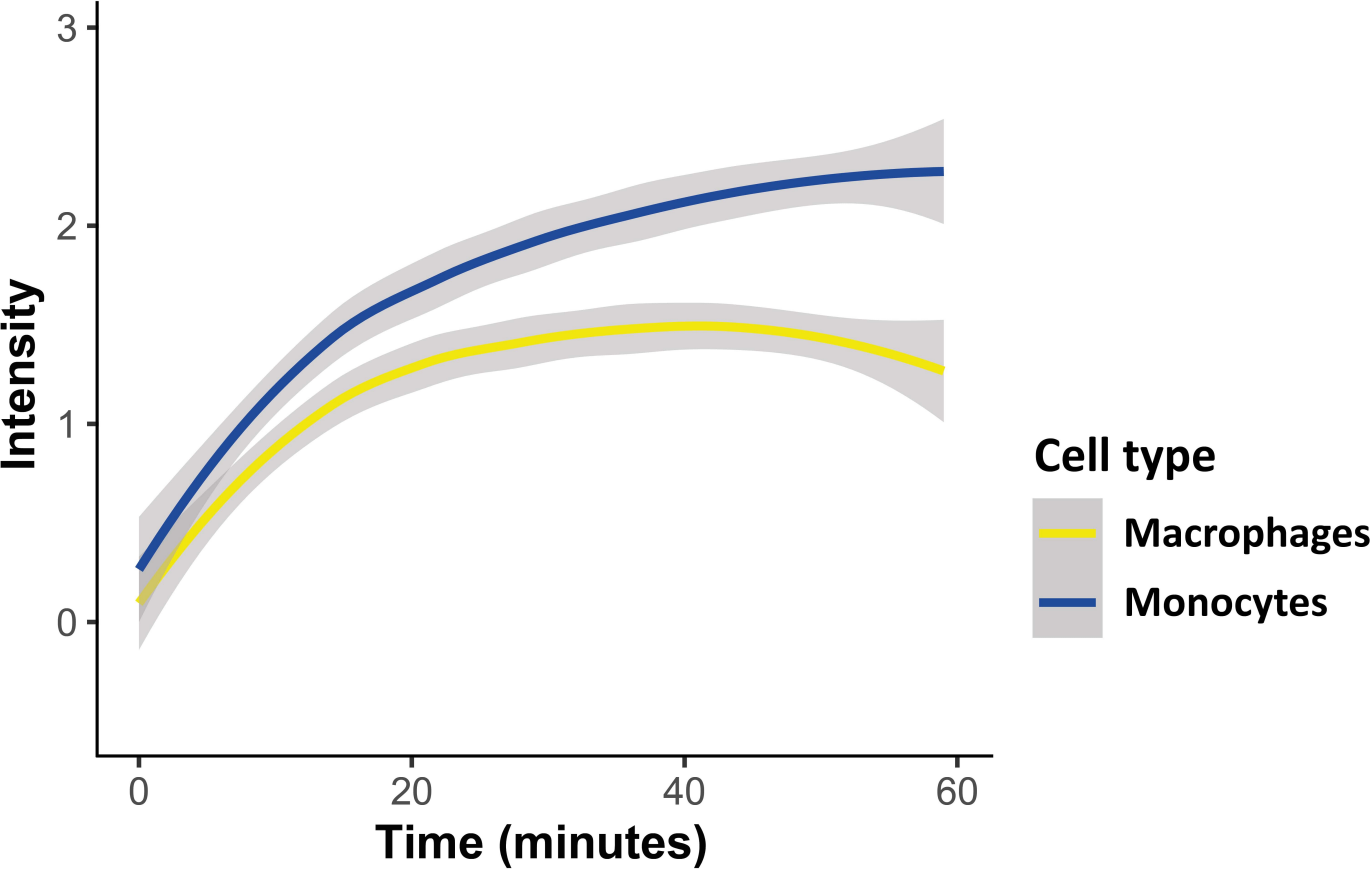
Kinetic measurement of *Pf*-derived EV uptake into either monocytes or macrophages using IFC. *Pf*-derived EVs were labeled by Thiazole orange (TO), and their uptake into monocytes (THP-1 cells) or macrophages (differentiated from THP-1 cells) was measured for 1 hour. Graph representing the signal modification in TO intensity that was detected over time originating from monocytes and macrophages recipient cells. Graph representing at least three independent biological replicates. Cell types were compared with a linear mixed effects model, with cell type and time as fixed factors, and sample ID as a random factor. Statistics were done in R, v. 4.0.4, using the package ‘lmerTest’. Grey area around the lines represents 95% confidence intervals, p<0.001.

As demonstrated, the intensity of the transferred RNA signal in both cell types increased over time, indicating progressive uptake of *Pf*-labeled EVs within both monocytes and macrophages, with the monocytes showing a higher intensity signal than the macrophages (**Figure 2**). Similar to monocytes, EVs are successfully internalized into human macrophages, evident by the increasing intensity rate over the course of 1 hour uptake. The acquired signal intensity in both cell types was normalized to the basal level signal received from the cells. RNA signal intensity in monocytes and macrophages, introduced with unlabeled EVs, did not change and remained below the threshold, as expected.

### *Pf*-Derived EV Uptake Into Monocytes and Macrophages Does Not Affect Cell Viability

We have previously performed a complementary viability assay using Trypan blue dye on recipient monocytes, following them for 72 hours post *Pf*-derived EVs uptake (Ofir-Birin et al., 2018). Our results showed that even after 24 hour of incubation with the EVs there was no significant difference in the percentages of the dead cells between untreated monocytes and monocytes treated with *Pf*-derived EVs. In both cases, the percentages of dead cells after 24 hours was around 2% (Ofir-Birin et al., 2018). In addition, we checked the percentages of apoptotic monocytes and macrophages using IFC, as previously described (Wortzel et al., 2017). Our result demonstrate that incubation of recipient monocytes and macrophages with *Pf*-derived EVs for 60 minutes causes a neglectable less than 1% of apoptotic cells (**Supplementary Figure 3**). Notably, excluding the apoptotic cells from the analysis didn’t affect the results.

### Area Threshold Feature Shows Distinct Distribution of the RNA Cargo in Macrophages Compared to Monocytes

**Figure 3:**
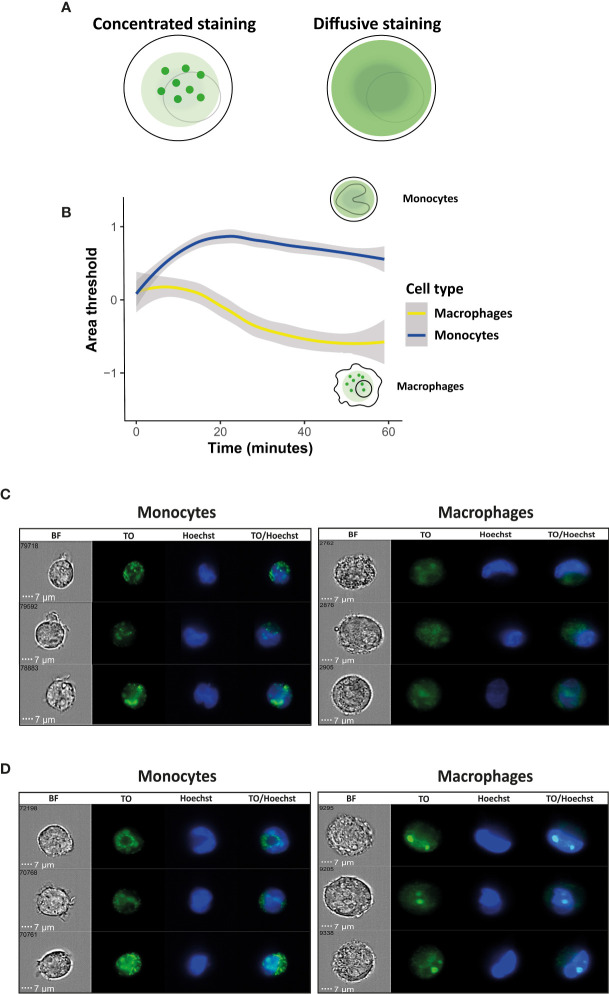
Kinetic measurement of the signal of EV RNA staining Area threshold inside recipient monocytes or macrophages. *Pf*-derived EVs were labeled by Thiazole orange (TO), and their uptake into monocytes and macrophages was measured for 1 hour. **(A)** Schematic representation of the Area Threshold feature. **(B)** Graph representing change in area threshold of the fluorescent signal inside the recipient cells over time. Cell types were compared with a linear mixed effects model, with cell type and time as fixed factors, and sample ID as a random factor. Statistics were done in R, v. 4.0.4, using the package ‘lmerTest’. For calculating changes relative to the starting point, a log2-fold change was calculated per each point, relative to its own sample’s average value of the first 1.5 minutes. Grey area around the lines represents 95% confidence intervals, p<0.001. **(C)** Signal detected by IFC from three representative recipient cells of monocytes and macrophages at the first 10 minutes of the uptake. **(D)** Signal detected by IFC from three representative recipient cells of monocytes and macrophages at 1 hour post uptake. BF-bright field, TO-thiazole orange, Hoechst-nuclear dye.

Once we confirmed that the parasitic-derived EVs are successfully internalized by both monocytes and macrophages, and we observed that the kinetic of the uptake was similar, we set up the system to examine whether there are any differences in the distribution pattern of the EV RNA cargo inside the two types of human host cells. To address that we measured the area of the RNA cargo distribution using the Area Threshold feature of the IFC, post uptake of EVs into the host recipient cells. First, a Threshold mask is created, that delineates the top 30% intensity pixels. Using this mask, the Area feature calculates the area (in square microns) of the 30% highest intensity pixels within the cells. Higher area values indicates that the staining texture is more diffuse across the cell, while lower area indicates a more ‘speckled’, concentrated staining (illustration, **Figure 3A**).

By using this feature, we were able to compare the data of the fluorescent signal of the RNA cargo distribution within a cell across the two different populations of host monocytes vs macrophages, both cells of different sizes. Interestingly, while the uptake kinetics over time were similar between monocytes and macrophages, we observed a significant difference between the ‘RNA cargo behaviors’ with the progress of the uptake, concentrated or diffusive, within these two host cells. As seen in **Figure 3B**, while it seems that the area of signal distribution is decreasing continuously over time in the macrophages (resulting in a more speckled and concentrated appearance) we observed the opposite trend in monocytes. It appears that in recipient monocytes the staining texture is diffusive at the early stage of the uptake and becoming more concentrated over the course of time. That could suggest an initial spread of the RNA cargo inside the cells, and a possibly concentration of it in a certain compartment/organelle as time goes by. In the recipient macrophages on the other hand, the fluorescent staining texture is becoming concentrated early on from the beginning of the uptake. Representative images from three individual recipient monocytes or macrophages at the time of 10 minutes and 1hour post internalization are shown in **Figures 3C** **, D**. These results might suggest a possible distinct role of the RNA cargo post uptake within monocytes vs macrophages.

### Max Contour Position Feature Shows Different Dynamic of Cargo Distribution in Macrophages Compared to Monocytes

**Figure 4:**
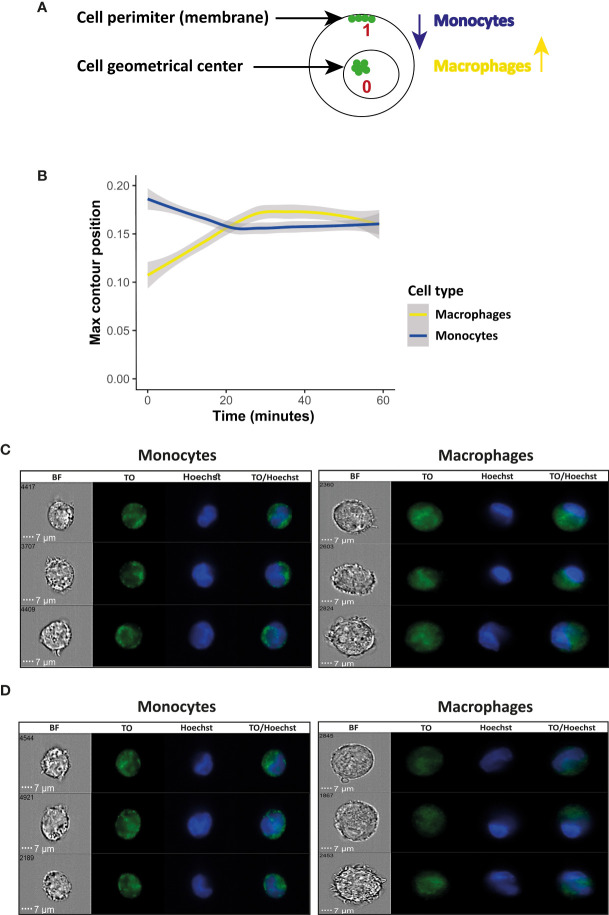
Kinetic measurement of fluorescent signal associated with RNA cargo using Max Contour Position inside recipient monocytes or macrophages. *Pf*-derived EVs were labeled by Thiazole orange (TO), and their uptake into monocytes (THP-1) cells and macrophages was measured for 1 hour. **(A)** Schematic representation of the Max Contour Position feature; 0 being the object center (the geometrical center of the cell) and 1 being the object perimeter (the cell membrane). **(B)** Graph representing the change in the subcellular location of the contour within the cell that has the highest intensity concentration, over time. Cell types were compared with a linear mixed effects model, with cell type and time as fixed factors, and sample ID as a random factor. Statistics were done in R, v. 4.0.4, using the package ‘lmerTest’. For calculating changes relative to the starting point, a log2-fold change was calculated per each point, relative to its own sample’s average value of the first 1.5 minutes. Grey area around the lines represents 95% confidence intervals, p<0.001. **(C)** Signal detected by IFC from three representative recipient cells of monocytes and macrophages at the first 10 minutes of the uptake. **(D)** Signal detected by IFC from three representative recipient cells of monocytes and macrophages at 1 hour post uptake. BF-bright field, TO-thiazole orange, Hoechst-nuclear dye.

Next, in order to measure the pattern of the RNA cargo distribution within monocytes as compared to macrophages, we used the Max Contour Position feature, which shows the location of the contour within the cell that has the highest intensity concentration. This feature is invariant to object size and can accommodate localized intensity concentrations. The actual location in the object is mapped to a number between 0 and 1, with 0 being the object center (the geometrical center of the cell) and 1 being the object perimeter the (cell membrane, illustration, **Figure 4A**). Again, we observed a significant difference in the pattern of the RNA cargo distribution in both cell types, in line with the results we obtained from the area threshold feature (**Figure 4B**). Representative images from three recipient monocytes and macrophages at the time points of post 10 minutes and 1hour of uptake are shown in **Figures 4C** **, D**. It is evident that while the final ‘regional point’ of the fluorescent signal post 1 hour of internalization is similar for both cells, the dynamic of the signal distribution is significantly different. While in macrophages the highest intensity concentration localizes towards the perimeter (cell membrane) over time, in the monocytes however the trend is opposite, localizes towards the center of the cell, suggesting that the RNA cargo might be concentrated towards the center, semi-nucleus, of the recipient cell over the course of its internalization (**Figure 4B**).

Taken together, these results might indicate that there are different modes of EV uptake for monocytes and macrophages. While both cells are part of the host immune system, the dynamics of the distribution of the signal of the EV RNA cargo in each is significantly different, suggesting that even subsets of cells from the immune system might utilize diverse mechanisms for the parasitic EV uptake. In addition, it could possibly point towards a distinct role of the RNA cargo in monocytes vs macrophages, post internalization.

Interestingly, as a complementary approach to uncover the role of the EV-RNA cargo in monocytes, we examined the localization of the RNA cargo within three different organelles (Nucleolus, Golgi and Lysosome) post uptake of the EVs to the recipient cells. Using RNA labeled-EVs and specific antibodies: anti-NPM1 for the Nucleolus subcellular localization (Holmberg Olausson et al., 2014), anti-Giantin for the Golgi subcellular localization (Schumann et al., 2020) and anti-LAMP2 for Lysosome subcellular localization (Szumska et al., 2019) we were able to detect co-localization of the TO staining signal of the RNA cargo with the NPM1 staining signal. Therefore our results suggest that there is a potentially co-localization of the EV-RNA cargo to the nucleolus but not to the other two cell organelles (**Supplementary Figure 4**).

## Discussion

There is an increasing accumulating evidence on the diverse roles of EVs as key mediators of cellular communication. EVs carry a range bioactive cargo, including nucleic acids and proteins, which can have a significant impact on the phenotype of their recipient cells. Recent data suggest that RNA cargo packed inside the EVs may have distinct roles in recipient cells, depending on the mode of EV internalization to the cells and their ability to recognize the RNA, using different cytosolic receptors (O’Brien et al., 2020).

In addition, EVs hold a potential for therapeutic applications, as their production is altered during different diseases (Mulcahy et al., 2014). It is therefore pivotal to uncover the mechanisms by which EVs are internalized by their target cells. Different endocytic pathways have been suggested for the uptake process, including clathrin-dependent endocytosis caveolin-mediated uptake, macropinocytosis, phagocytosis, and lipid raft-mediated internalization (Mulcahy et al., 2014; Anakor et al., 2021). Yet, so far the exact mechanism of the EV uptake is mostly unknown.

Current measurements of fluorescently labeled particles using conventional fluorescence microscopy are challenging due to EV fluid dispersal and small size. Since the vesicle size ranges between 50-200 nm, it places them below the diffraction limit of visible light and within the electronic noise (Ortyn et al., 2006; Erdbrügger et al., 2014; Vorobjev and Barteneva, 2016). Moreover, as the signal is summed for the whole cell, individual vesicles would be very difficult to detect both independently and within much larger cells. Thus, it is of a great need to develop an advanced method to track these nano-particles in a large-scale level and to visualize them along time post uptake within different target cells.

We have previously used IFC to monitor the EV cargo internalization into monocytes and to investigate the molecular effect of *Pf*-derived EV in host immune cells (Sisquella et al., 2017; Ofir-Birin et al., 2018). Here, we utilized IFC to follow live uptake of EVs into two different immune cells; monocytes and macrophages. IFC combines the advantages of speed and high-throughput quantification of flow cytometry, with the spatial high-resolution details acquired by microscopy. This allowed us to detect in real time, the uptake of EV-RNA-stained into living cells, and to quantify the kinetics of the fluorescent signal distribution and cellular localization post uptake.

Our measurements may hint different properties of the cargo distribution dynamics inside these recipient host cells (**Figures 2**, **3**, **4**). Interestingly, while the kinetic of the uptake was similar, we observed a significant difference in the signal distribution pattern, showing an opposite trend in its pixel intensity distribution and the dynamic of cellular localization over time (**Figures 2** **–** **4**).

We found that in the monocytes the staining texture is diffusive at the beginning of the uptake and becoming more concentrated over the course of time. In the macrophages on the other side, the area of the signal distribution is decreasing continuously over time. In addition, it appears that in macrophages the highest intensity concentration localizes towards the cell membrane over time, while in the monocytes the trend is the opposite, suggesting that the RNA cargo may localize towards the center of the recipient cell, over time.

These surprising results can be attributed to several reasons: I) possibly different uptake mechanism of EVs in monocytes vs macrophages, even though both cells are part of the same branch of innate immunity cells (mononuclear phagocyte system). II) Might be due to different mechanisms of cargo release post internalization within the cells. III) These data might also suggest that EV-RNA cargo facilitate distinct functions post uptake, for example binding to different cytosolic receptors or even co-localizing to a specific organelle inside the cells.

Indeed, we further followed this idea and used specific antibodies for staining different cellular organelles (Nucleolus, Golgi and Lysosome) (**Supplementary Figure 4**). Our data demonstrate a co-localization between the EV-RNA TO dye and the NPM1 nucleolar dye, suggesting a co-localization of the RNA cargo to the nucleolus rather than the Golgi or the Lysosome (**Supplementary Figure 4**).

Overall, we demonstrate that IFC can be applied as a robust tool to study different properties of the EV uptake and RNA cargo distribution. This approach could pave the way not only to measuring the process of vesicle internalization by different recipient cells, but also to directly studying activated protein movement and, thus, further investigation of related cellular signaling events. Characterizing the EV content by different dyes, tracking the kinetics of EV uptake into target cells and, finally, tracking the sub-cellular distribution of specific EV cargo within target cells may add another layer on the function of EVs in host–pathogen communication.

## Data Availability Statement

The raw data supporting the conclusions of this article will be made available by the authors, without undue reservation.

## Author Contributions

DA, HB, OD, and CJ performed the experiments. RR performed the statistical analysis. DA and HB analyzed the data. DA, HB, NR-R, and ZP wrote the manuscript. NR-R and ZP conceptualized and supervised the work. All authors contributed to the article and approved the submitted version.

## Funding

Weizmann Institute of Science, Staff Scientists Internal Grant (Awarded to ZP). The research of NR-R is supported by the Benoziyo Endowment Fund for the Advancement of Science, the Jeanne and Joseph Nissim Foundation for Life Sciences Research and the Samuel M. Soref and Helene K. Soref Foundation. NR-R is supported by a research grant from David E. and Sheri Stone and by a research grant from Richard and Mica Hadar. NR-R is the incumbent of the Enid Barden and Aaron J. Jade President’s Development Chair for New Scientists in Memory of Cantor John Y. Jade. NR-R is grateful for the support from the European Research Council (ERC) under the European Union’s Horizon 2020 research and innovation program (grant agreement No. 757743), the Israel Science Foundation (ISF) (619/16 and 2235/16) and the Israel Precision Medicine Program (IPMP) Research Grant Application no. 1637/20.

## Conflict of Interest

The authors declare that the research was conducted in the absence of any commercial or financial relationships that could be construed as a potential conflict of interest.

## Publisher’s Note

All claims expressed in this article are solely those of the authors and do not necessarily represent those of their affiliated organizations, or those of the publisher, the editors and the reviewers. Any product that may be evaluated in this article, or claim that may be made by its manufacturer, is not guaranteed or endorsed by the publisher.

## Supplementary Material

Supplementary Figure 1 Characterization of monocyte-derived macrophages after PMA treatment using IFC. Monocyte derived macrophages were compared to untreated monocytes (THP-1) and analyzed using IFC. **(A)** Graph representing the area of the cells **(B)** Monocytes and monocyte-derived macrophages were stained using anti CD14 antibody. Graph representing cells positive to CD14 surface marker. **(C)** Signal detected by IFC from two representative recipient cells of monocytes and macrophages. At least 5×10^4^ cells were collected from each sample.

Supplementary Figure 2 Characterization of *Pf*-derived EVs using NTA Nanosight measurement. *Pf*-derived EVs analyzed by Nanosight NS300 for size distribution and particle concentration. The graph represents the mean of 5×60 s measurements by Nanosight NS300. EV concertation is 5.65×10^8^ ± 6.7×10^7^ and the diameter mean is 106.2 nm. Representative results from at least three experiments are shown.

Supplementary Figure 3 Recipient cell viability following *Pf*-derived EV uptake. *Pf*-derived EVs were labeled by Thiazole orange (TO) and their uptake into monocytes (THP-1 cells) or macrophages (differentiated from THP-1 cells) was measured for 1 hour. **(A)** Graphs and tables below show the percentage of apoptotic cells following EV uptake. **(B)** Signal detected by IFC from two representative recipient cells of non-apoptotic monocytes and macrophages at 1 hour post EV uptake. **(C)** Signal detected by IFC from two representative recipient cells of apoptotic monocytes and macrophages at 1 hour post uptake. BF-bright field, TO-thiazole orange, Hoechst-nuclear dye.

Supplementary Figure 4 Co-localization of Thiazole Orange-labeled RNA cargo to the nucleolus. *Pf*-derived EVs were labeled by Thiazole orange (TO), and incubated with monocytes cells for 45 minutes. EV-treated cells were fixated, permeabilized and stained for different cellular organelles (Nucleolus, Lysosome and Golgi) using specific antibodies: mouse anti-NPM1 for the Nucleolus, mouse anti-LAMP2 for the Lysosome and rabbit anti-Giantin for the Golgi. Alexa Fluor 647-labeled donkey anti-rabbit IgG and Cy3 labeled donkey anti-mouse IgG were used as secondary antibodies. Cells were analyzed using IFC. Representative results from at least three independent experiments are shown. At least 5×10^4^ cells were collected from each sample. BF-bright field, TO-thiazole orange, NPM1-Nucleolus dye, Giantin- Golgi dye LAMP2-lysosomal dye, Hoechst-nuclear dye.
